# Supplementary material for: Curcumin Sensitises Cancerous Kidney Cells to TRAIL Induced Apoptosis via Let-7C Mediated Deregulation of Cell Cycle Proteins and Cellular Metabolism
Source: Int J Mol Sci. 2022 Aug 24;23(17):9569. doi: 10.3390/ijms23179569 (PMC9455736; doi:10.3390/ijms23179569)
Supplement: Supplementary file 1 [file ijms-23-09569-s001.zip › Supplementary file 0.2.pptx]

## Slide 1
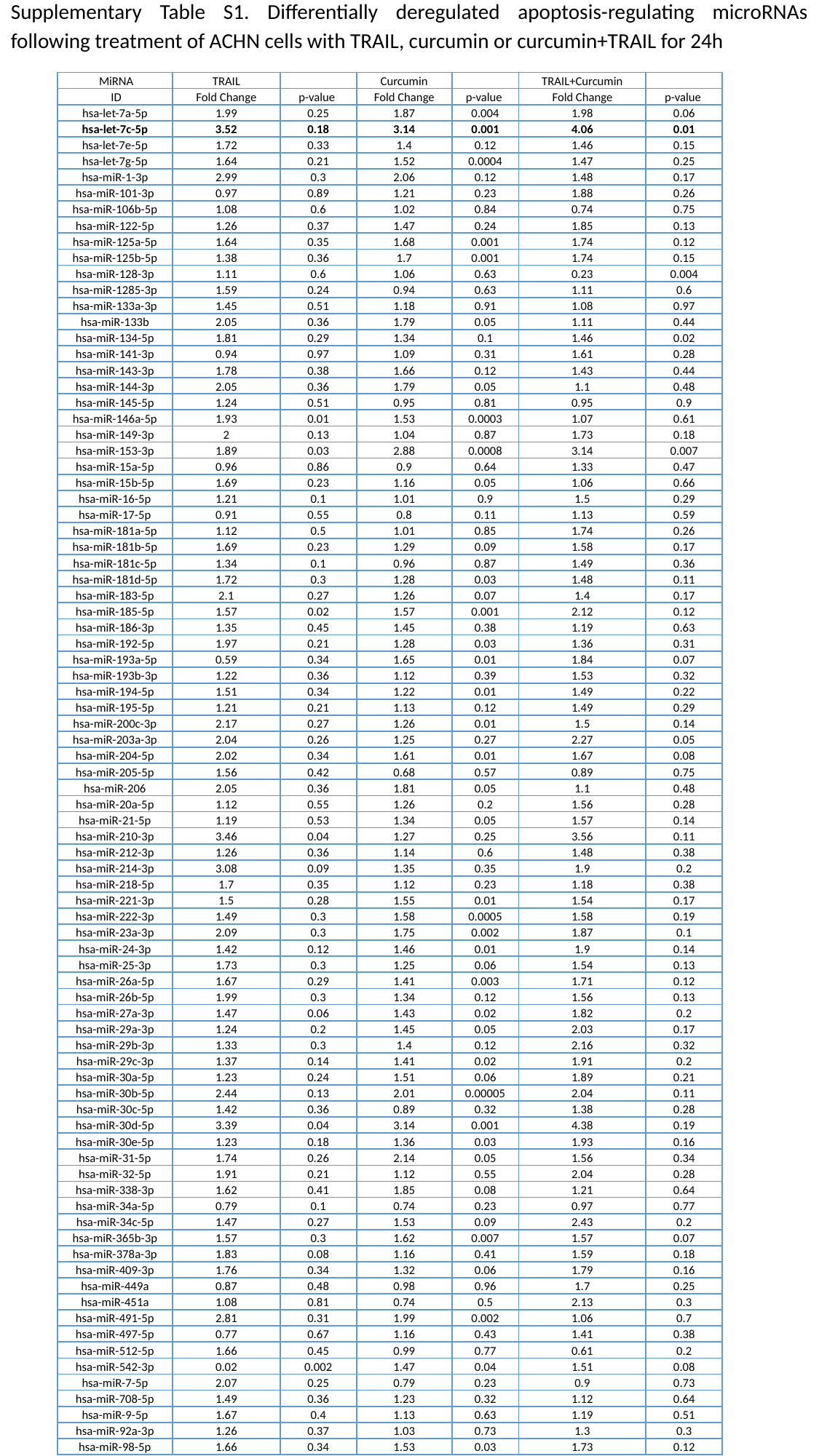

Supplementary Table S1. Differentially deregulated apoptosis-regulating microRNAs following treatment of ACHN cells with TRAIL, curcumin or curcumin+TRAIL for 24h
| MiRNA | TRAIL | | Curcumin | | TRAIL+Curcumin | |
| --- | --- | --- | --- | --- | --- | --- |
| ID | Fold Change | p-value | Fold Change | p-value | Fold Change | p-value |
| hsa-let-7a-5p | 1.99 | 0.25 | 1.87 | 0.004 | 1.98 | 0.06 |
| hsa-let-7c-5p | 3.52 | 0.18 | 3.14 | 0.001 | 4.06 | 0.01 |
| hsa-let-7e-5p | 1.72 | 0.33 | 1.4 | 0.12 | 1.46 | 0.15 |
| hsa-let-7g-5p | 1.64 | 0.21 | 1.52 | 0.0004 | 1.47 | 0.25 |
| hsa-miR-1-3p | 2.99 | 0.3 | 2.06 | 0.12 | 1.48 | 0.17 |
| hsa-miR-101-3p | 0.97 | 0.89 | 1.21 | 0.23 | 1.88 | 0.26 |
| hsa-miR-106b-5p | 1.08 | 0.6 | 1.02 | 0.84 | 0.74 | 0.75 |
| hsa-miR-122-5p | 1.26 | 0.37 | 1.47 | 0.24 | 1.85 | 0.13 |
| hsa-miR-125a-5p | 1.64 | 0.35 | 1.68 | 0.001 | 1.74 | 0.12 |
| hsa-miR-125b-5p | 1.38 | 0.36 | 1.7 | 0.001 | 1.74 | 0.15 |
| hsa-miR-128-3p | 1.11 | 0.6 | 1.06 | 0.63 | 0.23 | 0.004 |
| hsa-miR-1285-3p | 1.59 | 0.24 | 0.94 | 0.63 | 1.11 | 0.6 |
| hsa-miR-133a-3p | 1.45 | 0.51 | 1.18 | 0.91 | 1.08 | 0.97 |
| hsa-miR-133b | 2.05 | 0.36 | 1.79 | 0.05 | 1.11 | 0.44 |
| hsa-miR-134-5p | 1.81 | 0.29 | 1.34 | 0.1 | 1.46 | 0.02 |
| hsa-miR-141-3p | 0.94 | 0.97 | 1.09 | 0.31 | 1.61 | 0.28 |
| hsa-miR-143-3p | 1.78 | 0.38 | 1.66 | 0.12 | 1.43 | 0.44 |
| hsa-miR-144-3p | 2.05 | 0.36 | 1.79 | 0.05 | 1.1 | 0.48 |
| hsa-miR-145-5p | 1.24 | 0.51 | 0.95 | 0.81 | 0.95 | 0.9 |
| hsa-miR-146a-5p | 1.93 | 0.01 | 1.53 | 0.0003 | 1.07 | 0.61 |
| hsa-miR-149-3p | 2 | 0.13 | 1.04 | 0.87 | 1.73 | 0.18 |
| hsa-miR-153-3p | 1.89 | 0.03 | 2.88 | 0.0008 | 3.14 | 0.007 |
| hsa-miR-15a-5p | 0.96 | 0.86 | 0.9 | 0.64 | 1.33 | 0.47 |
| hsa-miR-15b-5p | 1.69 | 0.23 | 1.16 | 0.05 | 1.06 | 0.66 |
| hsa-miR-16-5p | 1.21 | 0.1 | 1.01 | 0.9 | 1.5 | 0.29 |
| hsa-miR-17-5p | 0.91 | 0.55 | 0.8 | 0.11 | 1.13 | 0.59 |
| hsa-miR-181a-5p | 1.12 | 0.5 | 1.01 | 0.85 | 1.74 | 0.26 |
| hsa-miR-181b-5p | 1.69 | 0.23 | 1.29 | 0.09 | 1.58 | 0.17 |
| hsa-miR-181c-5p | 1.34 | 0.1 | 0.96 | 0.87 | 1.49 | 0.36 |
| hsa-miR-181d-5p | 1.72 | 0.3 | 1.28 | 0.03 | 1.48 | 0.11 |
| hsa-miR-183-5p | 2.1 | 0.27 | 1.26 | 0.07 | 1.4 | 0.17 |
| hsa-miR-185-5p | 1.57 | 0.02 | 1.57 | 0.001 | 2.12 | 0.12 |
| hsa-miR-186-3p | 1.35 | 0.45 | 1.45 | 0.38 | 1.19 | 0.63 |
| hsa-miR-192-5p | 1.97 | 0.21 | 1.28 | 0.03 | 1.36 | 0.31 |
| hsa-miR-193a-5p | 0.59 | 0.34 | 1.65 | 0.01 | 1.84 | 0.07 |
| hsa-miR-193b-3p | 1.22 | 0.36 | 1.12 | 0.39 | 1.53 | 0.32 |
| hsa-miR-194-5p | 1.51 | 0.34 | 1.22 | 0.01 | 1.49 | 0.22 |
| hsa-miR-195-5p | 1.21 | 0.21 | 1.13 | 0.12 | 1.49 | 0.29 |
| hsa-miR-200c-3p | 2.17 | 0.27 | 1.26 | 0.01 | 1.5 | 0.14 |
| hsa-miR-203a-3p | 2.04 | 0.26 | 1.25 | 0.27 | 2.27 | 0.05 |
| hsa-miR-204-5p | 2.02 | 0.34 | 1.61 | 0.01 | 1.67 | 0.08 |
| hsa-miR-205-5p | 1.56 | 0.42 | 0.68 | 0.57 | 0.89 | 0.75 |
| hsa-miR-206 | 2.05 | 0.36 | 1.81 | 0.05 | 1.1 | 0.48 |
| hsa-miR-20a-5p | 1.12 | 0.55 | 1.26 | 0.2 | 1.56 | 0.28 |
| hsa-miR-21-5p | 1.19 | 0.53 | 1.34 | 0.05 | 1.57 | 0.14 |
| hsa-miR-210-3p | 3.46 | 0.04 | 1.27 | 0.25 | 3.56 | 0.11 |
| hsa-miR-212-3p | 1.26 | 0.36 | 1.14 | 0.6 | 1.48 | 0.38 |
| hsa-miR-214-3p | 3.08 | 0.09 | 1.35 | 0.35 | 1.9 | 0.2 |
| hsa-miR-218-5p | 1.7 | 0.35 | 1.12 | 0.23 | 1.18 | 0.38 |
| hsa-miR-221-3p | 1.5 | 0.28 | 1.55 | 0.01 | 1.54 | 0.17 |
| hsa-miR-222-3p | 1.49 | 0.3 | 1.58 | 0.0005 | 1.58 | 0.19 |
| hsa-miR-23a-3p | 2.09 | 0.3 | 1.75 | 0.002 | 1.87 | 0.1 |
| hsa-miR-24-3p | 1.42 | 0.12 | 1.46 | 0.01 | 1.9 | 0.14 |
| hsa-miR-25-3p | 1.73 | 0.3 | 1.25 | 0.06 | 1.54 | 0.13 |
| hsa-miR-26a-5p | 1.67 | 0.29 | 1.41 | 0.003 | 1.71 | 0.12 |
| hsa-miR-26b-5p | 1.99 | 0.3 | 1.34 | 0.12 | 1.56 | 0.13 |
| hsa-miR-27a-3p | 1.47 | 0.06 | 1.43 | 0.02 | 1.82 | 0.2 |
| hsa-miR-29a-3p | 1.24 | 0.2 | 1.45 | 0.05 | 2.03 | 0.17 |
| hsa-miR-29b-3p | 1.33 | 0.3 | 1.4 | 0.12 | 2.16 | 0.32 |
| hsa-miR-29c-3p | 1.37 | 0.14 | 1.41 | 0.02 | 1.91 | 0.2 |
| hsa-miR-30a-5p | 1.23 | 0.24 | 1.51 | 0.06 | 1.89 | 0.21 |
| hsa-miR-30b-5p | 2.44 | 0.13 | 2.01 | 0.00005 | 2.04 | 0.11 |
| hsa-miR-30c-5p | 1.42 | 0.36 | 0.89 | 0.32 | 1.38 | 0.28 |
| hsa-miR-30d-5p | 3.39 | 0.04 | 3.14 | 0.001 | 4.38 | 0.19 |
| hsa-miR-30e-5p | 1.23 | 0.18 | 1.36 | 0.03 | 1.93 | 0.16 |
| hsa-miR-31-5p | 1.74 | 0.26 | 2.14 | 0.05 | 1.56 | 0.34 |
| hsa-miR-32-5p | 1.91 | 0.21 | 1.12 | 0.55 | 2.04 | 0.28 |
| hsa-miR-338-3p | 1.62 | 0.41 | 1.85 | 0.08 | 1.21 | 0.64 |
| hsa-miR-34a-5p | 0.79 | 0.1 | 0.74 | 0.23 | 0.97 | 0.77 |
| hsa-miR-34c-5p | 1.47 | 0.27 | 1.53 | 0.09 | 2.43 | 0.2 |
| hsa-miR-365b-3p | 1.57 | 0.3 | 1.62 | 0.007 | 1.57 | 0.07 |
| hsa-miR-378a-3p | 1.83 | 0.08 | 1.16 | 0.41 | 1.59 | 0.18 |
| hsa-miR-409-3p | 1.76 | 0.34 | 1.32 | 0.06 | 1.79 | 0.16 |
| hsa-miR-449a | 0.87 | 0.48 | 0.98 | 0.96 | 1.7 | 0.25 |
| hsa-miR-451a | 1.08 | 0.81 | 0.74 | 0.5 | 2.13 | 0.3 |
| hsa-miR-491-5p | 2.81 | 0.31 | 1.99 | 0.002 | 1.06 | 0.7 |
| hsa-miR-497-5p | 0.77 | 0.67 | 1.16 | 0.43 | 1.41 | 0.38 |
| hsa-miR-512-5p | 1.66 | 0.45 | 0.99 | 0.77 | 0.61 | 0.2 |
| hsa-miR-542-3p | 0.02 | 0.002 | 1.47 | 0.04 | 1.51 | 0.08 |
| hsa-miR-7-5p | 2.07 | 0.25 | 0.79 | 0.23 | 0.9 | 0.73 |
| hsa-miR-708-5p | 1.49 | 0.36 | 1.23 | 0.32 | 1.12 | 0.64 |
| hsa-miR-9-5p | 1.67 | 0.4 | 1.13 | 0.63 | 1.19 | 0.51 |
| hsa-miR-92a-3p | 1.26 | 0.37 | 1.03 | 0.73 | 1.3 | 0.3 |
| hsa-miR-98-5p | 1.66 | 0.34 | 1.53 | 0.03 | 1.73 | 0.12 |

## Slide 2
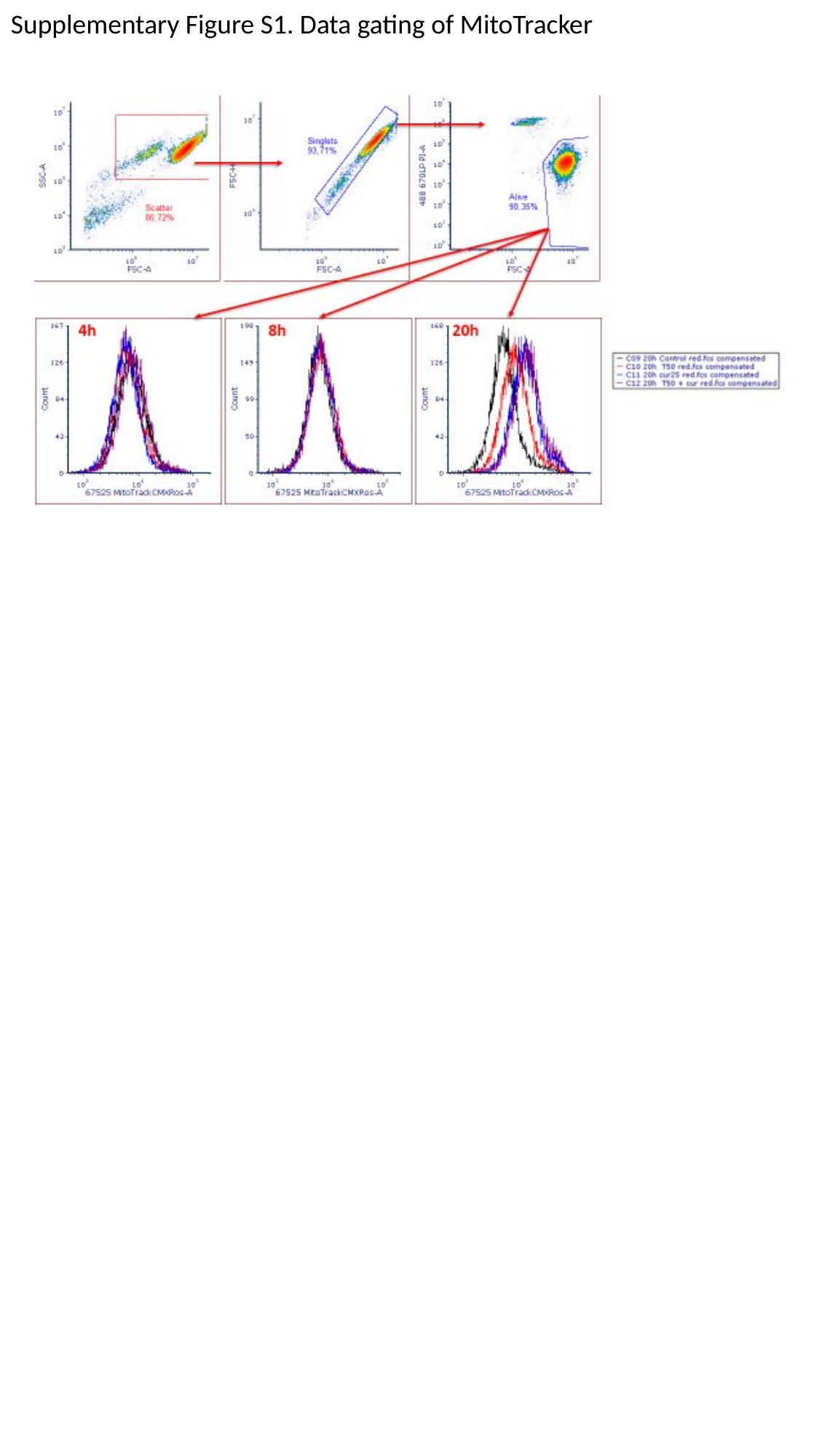

Supplementary Figure S1. Data gating of MitoTracker

## Slide 3
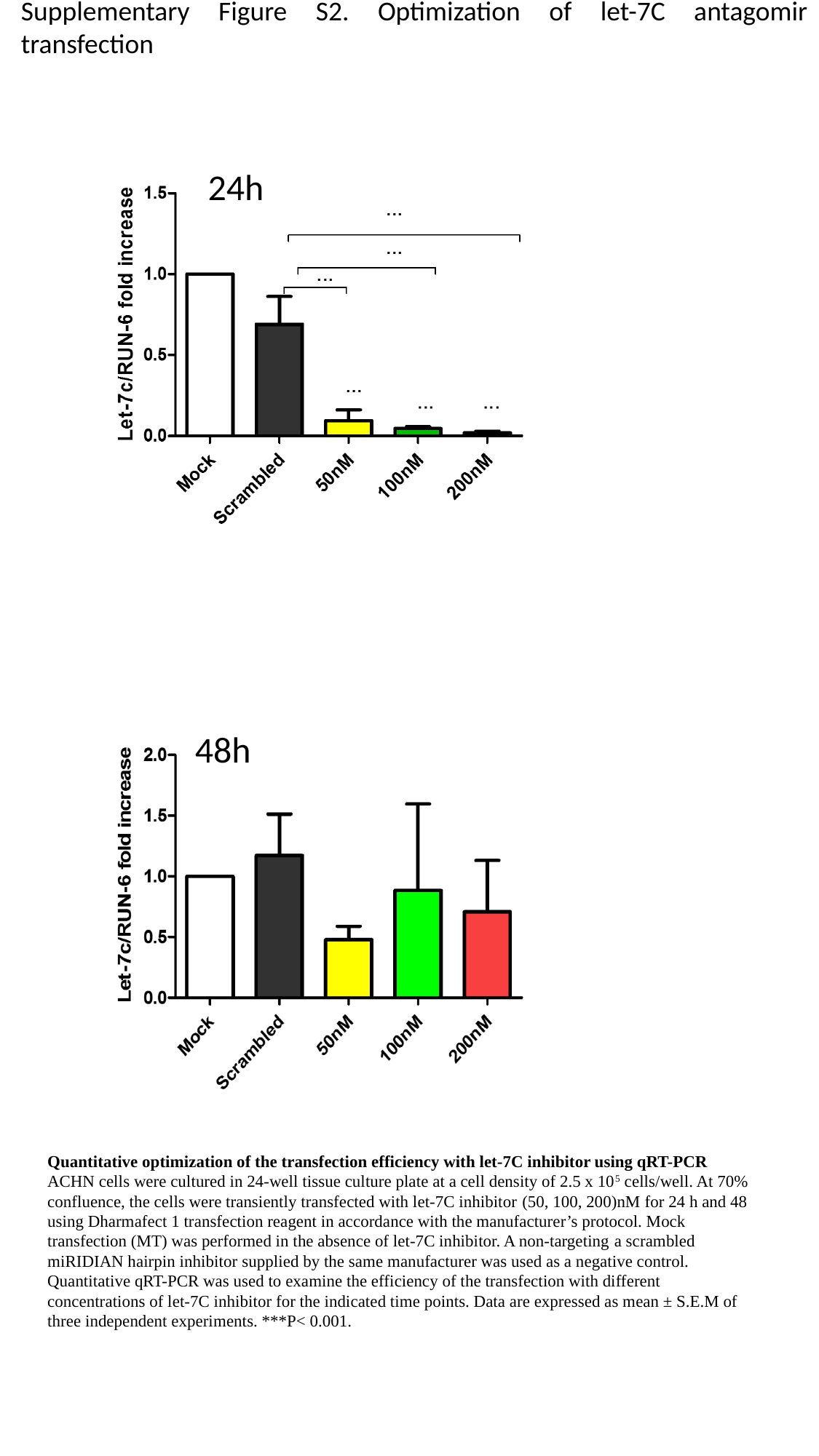

Supplementary Figure S2. Optimization of let-7C antagomir transfection
24h
48h
Quantitative optimization of the transfection efficiency with let-7C inhibitor using qRT-PCR
ACHN cells were cultured in 24-well tissue culture plate at a cell density of 2.5 x 10^5^ cells/well. At 70% confluence, the cells were transiently transfected with let-7C inhibitor (50, 100, 200)nM for 24 h and 48 using Dharmafect 1 transfection reagent in accordance with the manufacturer’s protocol. Mock transfection (MT) was performed in the absence of let-7C inhibitor. A non-targeting a scrambled miRIDIAN hairpin inhibitor supplied by the same manufacturer was used as a negative control. Quantitative qRT-PCR was used to examine the efficiency of the transfection with different concentrations of let-7C inhibitor for the indicated time points. Data are expressed as mean ± S.E.M of three independent experiments. ***P< 0.001.

## Slide 4
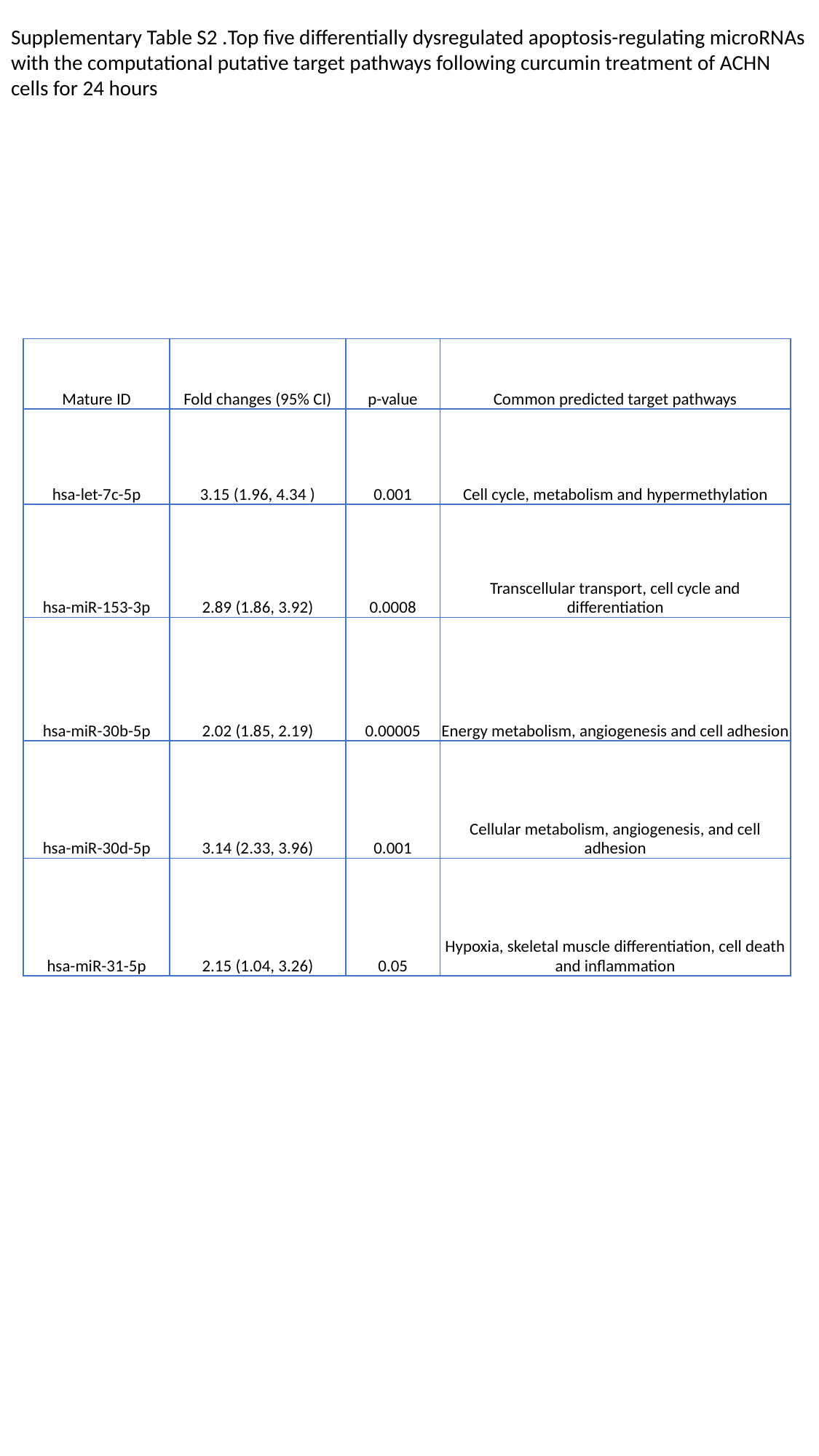

Supplementary Table S2 .Top five differentially dysregulated apoptosis-regulating microRNAs with the computational putative target pathways following curcumin treatment of ACHN cells for 24 hours
| Mature ID | Fold changes (95% CI) | p-value | Common predicted target pathways |
| --- | --- | --- | --- |
| hsa-let-7c-5p | 3.15 (1.96, 4.34 ) | 0.001 | Cell cycle, metabolism and hypermethylation |
| hsa-miR-153-3p | 2.89 (1.86, 3.92) | 0.0008 | Transcellular transport, cell cycle and differentiation |
| hsa-miR-30b-5p | 2.02 (1.85, 2.19) | 0.00005 | Energy metabolism, angiogenesis and cell adhesion |
| hsa-miR-30d-5p | 3.14 (2.33, 3.96) | 0.001 | Cellular metabolism, angiogenesis, and cell adhesion |
| hsa-miR-31-5p | 2.15 (1.04, 3.26) | 0.05 | Hypoxia, skeletal muscle differentiation, cell death and inflammation |
